# Reply to letter from Drs. Piotr Kanclerz and Andrzej Grzybowski entitled “Glistenings might be associated with disability glare”

**DOI:** 10.1177/11206721211059004

**Published:** 2021-11-10

**Authors:** Nick Stanojcic, Christopher Hull, David P.S. O’Brart

**Affiliations:** 1 Department of Ophthalmology, 8945 Guy’s and St Thomas’ NHS Foundation Trust, London, UK; 2 King’s College London, London, UK; 3 School of Health Science, Division of Optometry and Visual Sciences, City University, London, UK

**Keywords:** glistenings, intraocular lens implants (IOLs)

We thank Drs. Kanclerz and Grzybowski for their interest in our work.

In our review, we extensively discussed the published literature pertaining to the effects of lens glistenings on visual function. We agree that forward light scatter from glistenings will cause reduced image contrast However, the key question is whether the forward light scatter is sufficient to cause disability glare. As noted by Drs Kanclerz and Grzybowski, laboratory models have demonstrated correlations between glistenings density and size and light scatter, including our own publication,^1^ but clinical studies reporting visual impairment with glistenings occurrence are in the minority and have limitations. Indeed, the retrospective cohort study of Henriksen et al.,^2^ referenced in the letter, was a small pilot study, lacking methodology relating to image acquisition and analysis, such as ambient illumination level and digital/analogue camera settings, and with glistenings quantification and analysis undertaken using operator-dependent, general purpose, image processing software (‘ImageJ’). In addition, the authors stated themselves, that the study was underpowered to detect the true effect.^2^

We would like to direct Drs. Kanclerz and Grzybowski to our recent paper, undertaken to further address such issues and which is referenced and discussed in the review.^3^ In a prospective methodology we evaluated forward light scatter in a cohort of patients with glistenings. Using a newly developed, defined, reproducible, standardized 8-point ordinal scale of glistenings density and an array of computerized visual function tests (Advanced Vision and Optometric Tests, City Occupational, London, UK), performed under strictly controlled ambient conditions and based on the same scientific principle as the C-Quant test (Oculus, Optigeräte, Germany), we found no association between glistening grades and visual function including straylight parameter or integrated straylight parameter.^3^ This study we feel further adds to the evidence that glistenings, unless present in extremely large amounts, have a minimal effect on visual performance in vivo.

We do, however, recognise the limitations of light scatter testing that is currently available to undertake such in vivo investigations. In their study Colin and Orignac could only obtain valid results in about 50% of cases with the C-quant test^4^ This same test was used by Henriksen et al. although no mention was made in their paper of the percentage of successfully completed tests.^2^ It is of note that in our own study.^3^ approximately 20% of participants were not able to complete our light scatter test It must also be recognized that evaluation of glistenings themselves and their visual perturbations in vivo is much more challenging than in vitro. In our experience, the precise quantification of glistenings in vivo has proven difficult and dependent on multiple factors often not discussed in studies, such as ambient illumination, slit lamp parameters and image acquisition parameters.

In our review, we have not asserted that lens glistenings do not cause intraocular light scatter, but that convincing in-vivo evidence does not yet exist The paper by Labuz et al.,^5^ cited by Drs Kanclerz and Grzybowski, demonstrated that to produce a straylight value equivalent to the 70-year-old crystalline lens would require 400 glistenings/mm^2^ of 15 micron diameter, a figure rising to 3000 glistenings/mm^2^ if they are of 5 micron diameter.^5^ These levels are rarely seen in modern clinical practice and support the balance of evidence that glistenings have a negligible effect on visual function at levels found in modern IOLs.

Regarding the comments concerning IOL calcification, Neuhann et al.^6^ in their excellent paper, which is cited and discussed in our review, proposed three possible routes for IOL calcification: primary calcification which is related to the IOL itself (e.g. the polymer, manufacturing or packaging process); secondary, that is not only dependent on the IOL but also associated with pre-existing disease, which may involve breakdown of blood aqueous barrier; and false positive calcification or pseudo-calcification that occurs due to misdiagnosis of tissue artefacts or incorrect use of special stains. We disagree that we only reviewed the risk factors for secondary calcification; we discussed and high-lighted in detail and cited what has been published in multiple publications and hypothesised regarding IOL calcification relating to the IOL itself including issues with polymers, manufacturing, and packaging.

## References

[1] Philippaki E O’Brart DP Hull CC . Comparison of glistenings formation and their effect on forward light scatter between the acrysof SN60WF and eternity natural Uni NW-60 intraocular lenses. BMJ Open Ophthalmology 2020; 5: e000399. 10.1136/bmjophth-2019-000399 PMC7044831 32154371

[2] Henriksen BS Kinard K Olson RJ . Effect of intraocular lens glistening size on visual quality. J Cataract Refract Surg 2015; 41: 1190–1198. 26189379 10.1016/j.jcrs.2014.09.051

[3] Stanojcic N O’Brart DPS Maycock N , et al. Effects of intraocular lens glistenings on visual function: a prospective study and presentation of a new glistenings grading methodology. BMJ Open Ophthalmol 2019; 4: e000266.. 10.1136/bmjophth-2018-000266 PMC6528773 31179397

[4] Colin J Orignac I . Glistenings on intraocular lenses in healthy eyes: effects and associations. J Refract Surg 2011; 27: 869–875. 21800784 10.3928/1081597X-20110725-01

[5] Labuz G Reus NJ van den Berg TJ . Straylight from glistenings in intraocular lenses: in vitro study. J Cataract Refract Surg 2017; 43: 102–108. 28317662 10.1016/j.jcrs.2016.10.027

[6] Neuhann IM Kleinmann G Apple DJ . A New classification of calcification of intraocular lenses. Ophthalmology 2008; 115: 73–79. 17498804 10.1016/j.ophtha.2007.02.016

